# Mast Cells in the Skin: Defenders of Integrity or Offenders in Inflammation?

**DOI:** 10.3390/ijms22094589

**Published:** 2021-04-27

**Authors:** Martin Voss, Johanna Kotrba, Evelyn Gaffal, Konstantinos Katsoulis-Dimitriou, Anne Dudeck

**Affiliations:** 1 Medical Faculty, Institute for Molecular and Clinical Immunology, Otto-Von-Guericke-University Magdeburg, 39120 Magdeburg, Germany; martin.voss@med.ovgu.de (M.V.); johanna.kotrba@med.ovgu.de (J.K.); konstantinos.katsoulis-dimitriou@med.ovgu.de (K.K.-D.); 2 Laboratory for Experimental Dermatology, Department of Dermatology, University Hospital Magdeburg, 39120 Magdeburg, Germany; evelyn.gaffal@med.ovgu.de; 3 Health Campus Immunology, Infectiology and Inflammation, Otto-Von-Guericke-University Magdeburg, 39120 Magdeburg, Germany

**Keywords:** mast cells, innate immunity, host defense, inflammatory skin disorders

## Abstract

Mast cells (MCs) are best-known as key effector cells of immediate-type allergic reactions that may even culminate in life-threatening anaphylactic shock syndromes. However, strategically positioned at the host–environment interfaces and equipped with a plethora of receptors, MCs also play an important role in the first-line defense against pathogens. Their main characteristic, the huge amount of preformed proinflammatory mediators embedded in secretory granules, allows for a rapid response and initiation of further immune effector cell recruitment. The same mechanism, however, may account for detrimental overshooting responses. MCs are not only detrimental in MC-driven diseases but also responsible for disease exacerbation in other inflammatory disorders. Focusing on the skin as the largest immune organ, we herein review both beneficial and detrimental functions of skin MCs, from skin barrier integrity via host defense mechanisms to MC-driven inflammatory skin disorders. Moreover, we emphasize the importance of IgE-independent pathways of MC activation and their role in sustained chronic skin inflammation and disease exacerbation.

## 1. Introduction

The skin is one of the body’s largest organs and serves as a barrier to the outer environment [1,2]. It consists of a complex network of various immune cell types and was, therefore, very early on identified as an immune organ [3]. Within the skin, immune cells are mainly located in the dermis [2] and are important for maintaining skin homeostasis and protecting against mechanical, chemical and pathogenic insults [2,4,5]. The skin is the organ richest in Mast cells (MC), with MCs constituting 10% of leukocytes in the mouse ear skin [6,7]. In healthy adult skin, more MCs are found in distal areas (arms and legs) compared to proximal areas [8]. MCs populate tissues as progenitors derived from the bone marrow or the yolk sac and differentiate locally into mature MCs in response to the existing cytokine micromilieu. Particularly in the skin, recent research in mice has shown that MCs are mainly of yolk sac origin [5,9,10,11,12]. Under physiologic conditions, MCs are located in the dermis, near blood vessels, nerves and hair follicles [6,13]. Perivascular MCs display an elongated cell shape compared to interstitial MCs and, in the case of arterioles, even line the vessel in parallel to the vessel axis [14,15]. Additionally, we recently demonstrated that a distinct number of perivascular MCs were not only attached to the vessel exterior. They formed intraluminal sheets, even under physiologic conditions, and constituted a part of the vascular unit directly in alignment with endothelial cells (ECs) [15].

MCs were first described in 1878 by Paul Ehrlich based on their unique staining with metachromatic dyes [16]. Indeed, MCs contain a large amount of secretory granules that are composed of a proteoglycan structure and in which, due to electrostatic interactions, a plethora of mediators is embedded, including MC-specific proteases, non-specific proteases, cytokines, chemokines, and growth factors [16]. Later on, it was recognized that MCs (1) express a high-density of the high-affinity IgE receptor, FcεRI, (2) bind soluble immunoglobulin E (IgE) antibodies, which are produced by B cells after sensitization to a specific allergen, and (3) are the main deposit of histamine, the bioactive amine, driving allergic reactions and anaphylaxis [17,18]. After reexposure, the same allergen is recognized by antigen-specific IgE, leading to crosslinking of the FcεR and intracellular Ca^2+^ release. This signaling cascade results in immediate degranulation of the secretory granules, from which, after exocytosis, mediators like histamine, cytokines and proteases extrude sequentially [17,18,19,20]. MC degranulation is followed by secretion of lipid mediators, including leukotrienes and prostaglandins, and de novo synthesis of a broad spectrum of cytokines, chemokines and growth factors [13]. Interestingly, IgE production by B cells is stimulated by MC degranulation, and, at the same time, FcεR expression on the MC plasma membrane is enhanced by IgE in a positive feedback loop [21].

Consequently, skin MCs are strategically positioned and equipped for the defense against invading pathogens, response to allergen encounters, and interaction with a resident or infiltrating immune effector cells. However, these interactions may be beneficial or detrimental, and MCs are heavily involved in inflammatory skin disorders. The purpose of this review is to delineate the fascinating role of MCs in skin barrier function, all the way from maintenance of skin homeostasis to infections and inflammatory disorders.

## 2. Role of Mast Cells in Barrier Integrity and Host Defense

### 2.1. Mast Cells in Skin Homeostasis

MCs have been reported to be very important as regulators of epidermal barrier function and skin homeostasis and play important roles in wound healing and skin aging [6,22,23,24,25,26,27]. Their role in skin homeostasis has been often reported, but so far, the underlying mechanisms could not be identified in detail. A common hypothesis is that MCs display their homeostatic effects through interactions with neighboring immune and non-immune-cells. Several publications underline the importance of complex intercellular communication in maintaining barrier function and immune homeostasis [4,6,28,29,30].

MCs are an important source of cytokines, chemokines and growth factors, which can play distinct roles in the skin barrier [31,32]. MCs do not only release mediators during degranulation in response to certain stimuli but they are also reported to secrete them constitutively, which serve as an important tool to communicate with neighboring cells [31].

Especially the communication with fibroblasts (FB) was of particular focus in the last decades [6,30]. FBs are also located in the dermis near MCs and are important for structural skin integrity [5,33]. MCs induce FB proliferation via interleukin (IL)-4 [34,35], IL-13 [36], vascular endothelial growth factor (VEGF) and basic fibroblast growth factor (bFGF) [6,37,38]. Moreover, bidirectional communication is necessary for maintaining skin-barrier homeostasis. The expression and secretion of stem cell factor (SCF), the MC growth factor, by FB promotes MC differentiation and controls MC activation [39,40]. Moreover, FBs inhibit MC activation by secreting the enzyme Cyp26b1, which locally downregulates P2X7 expression on skin MCs. This mechanism serves as a unique skin-barrier homeostatic network inhibiting ATP-dependent MC activation [30]. Additionally, MCs continuously secrete tumor necrosis factor (TNF) [36], IL-1β [41,42], IL-4 [43,44,45,46,47], bFGF/FGF-2 [48,49,50], transforming growth factor β1 (TGF-β1) [51] and VEGF/VPF [52,53]. These mediators have recently been also reported to influence FB functions [6,34,36,54,55], but the direct MC–FB interaction via these cytokines remains to be proven. In addition to their interactions with FBs, MCs were also reported to contribute to skin homeostasis via interactions with keratinocytes (KC) [5,28]. KCs play an important role in MC maturation since they also produce SCF [56,57]. On the other hand, MCs are reported to have both inhibitory and activating effects on KCs. For example, they can express keratinocyte growth factor (KGF) [58] and platelet-activating factor (PAF) [28,59] that activate KCs, while MC release of histamine, heparin and other MC mediators inhibits KC proliferation and, therefore, controls epidermal regeneration [60,61]. MC tryptase and chymase are reported to promote FB proliferation while inhibiting KC proliferation [62]. Additionally, Sehra et al. could show that MCs can regulate epidermal differentiation complex (EDC) genes, suggesting a protective role of MCs in regulating epidermal barrier integrity. Mice lacking MCs exhibited decreased levels of EDC gene expression, which was associated with higher permeability for environmental antigens [63].

Finally, MCs can act on ECs to maintain skin homeostasis [5,28]. Angiogenesis is an important process for normal skin development, homeostasis, and remodeling [64,65]. Skin MCs can spontaneously secrete several angiogenesis-related factors and, therefore, exhibit an intrinsic role in vascular development [28,66,67]. MC-derived tryptase additionally promotes angiogenesis by degrading the basement membrane [68]. Among all vasoactive mediators that are released by MCs [59], it was reported that MCs impact on blood ECs (BECs) via histamine [69], TNF [70,71], leukotrienes [72], prostaglandin D2 (PGD2) [73] PAF [74], VEGF-A and VEGF-B [65,75], IL-13 [76] and IL-1β [77] and on lymphatic ECs (LECs) via histamine [78] VEGF-C and VEGF-D [68,75]. However, these interactions are also characterized by a bidirectional mode. MCs are not only a source but also a target of angiogenic and lymphangiogenic factors [68,75]. VEGF-A that is expressed by ECs can regulate MC proliferation and maturation within the skin [79].

Collectively, the homeostatic environment of the skin is regulated very precisely, and MCs play a crucial role in maintaining skin barrier homeostasis and integrity by interacting with neighboring non-immune cells, like FBs, KCs and ECs [58].

### 2.2. Mast Cells as a Link between Innate and Adaptive Immunity

MCs are mainly known for their key effector functions in type I allergy, where they are activated by crosslinking of cell-surface-bound FcεRI–IgE complexes by specific antigen [80,81,82]. Additionally, MCs are known to exhibit important innate and adaptive immunity functions, as we previously discussed [83]. Given their response repertoire, consisting of a wide range of surface receptors and proinflammatory mediators, and their strategic positioning, MCs contribute to the first line of host defense against invading pathogens [80,82,84,85,86]. MCs express a broad spectrum of pattern recognition receptors, including Toll-like receptors (TLRs), although very little TLR expression was observed in human skin MCs [87,88], Fc receptors and complement receptors [89]. Additionally, MCs can sense cell stress and tissue damage through alarmin and purinergic receptors and be activated or modulated by binding cytokines, growth factors, chemokines and neuropeptides [84,90,91,92,93].

One of the most characteristic features of MCs is their high amount of intracellular secretory granules, which contain a plethora of preformed mediators [16]. Upon external stimuli, MCs can degranulate within seconds, which allows a faster response than other tissue-resident immune cells [94,95,96,97]. Therefore, in many cases, MCs act as initiators of immune responses. As explained above, MC granules contain a wide range of preformed mediators, including histamine, cytokines, chemokines and proteases [16,98]. They immediately release bioactive amines, histamine and serotonin [99] and trigger blood vessel dilatation and permeabilization, finally causing edema formation. These vascular responses are further enhanced by TNF, proteases and eicosanoids that activate vascular ECs [100]. Subsequently, MCs initiate early neutrophil (Nph) recruitment, for example, by TNF, particularly by direct degranulation of TNF into the bloodstream leading to priming of circulating Nph, by secretion of Nph attractants, such as CXCL-1 (KC) and CXCL-2 (MIP-2), and by the release of IL-33 [70,101,102,103]. Moreover, MCs have been reported to enhance Nph effector functions [104,105]. Due to causing increased vascular permeability and edema formation, MCs may also impact on recruitment of other innate and adaptive immune cells to the site of infection or inflammation [67].

In addition to their innate functions that foster adaptive immune responses, MCs can indirectly affect adaptive immunity by modulating dendritic cell (DC) functions [106,107]. MCs and DCs reside near environmental interfaces, allowing for intense intercellular communication [106,108]. This communication can be based on soluble MC mediators, such as histamine and TNF, or on uptake of intact MC granules by DCs and promotes DC migration, DC maturation and T cell priming capacity [109,110]. Moreover, direct MC–DC interactions, including synapse formation, modulate DC functions and thereby fine-tune adaptive immunity [111,112]. We could recently show that MC–DC synapse formation culminates in MHC class II transfer from DCs to MCs, thereby equipping MCs with antigen-presentation capacities that may contribute to effector T cell activation [112].

Inline, the antigen-presenting capacity of MCs has been reported in several studies [91,113,114]. Direct MC–T cell interaction and synapse formation included MHC class II and costimulatory molecules (CD80 and CD86) but can also be mediated by endothelial cell protein C receptor (EPCR) or MHC class I [114,115,116,117]. Additionally, MCs modulate T cell functions by releasing exosomes and soluble mediators. The mode of MC stimulation can either promote T cell polarization towards T_H_1, T_H_2 or T_H_17 or control immune responses by T_reg_ activation via IL-2 or by direct inhibition of effector T cells via IL-10 [117,118,119,120,121,122,123,124].

Collectively, as discussed in more detail in Katsoulis-Dimitriou et al. [83], MCs critically contribute to innate host defense but also link the innate and adaptive immune response.

### 2.3. The Role of Mast Cells in Venom Detoxification

In 1991 already, Margie Profet hypothesized that allergic responses might be beneficial in the defense against venoms [125]. However, it took more than 20 years until this theory could be confirmed. By studying mice sensitized with a sub-lethal dose of honeybee venom, Marichal et al. showed that IgE antibody binding to FcεRI was responsible for conferring protection against subsequent lethal challenge [126]. Consequently, IgE-dependent MC activation, the mode of allergic reactions [81], has a protective role against noxious substances [127]. Importantly, a protective effect of MCs against venoms from honeybees and snakes, as well as wasps, scorpions, and the Gila monster, has been reported [126,128]. The response to poison from the *Thalassophryne natteteri* toadfish has also been recently shown to be IgE mediated [129]. Moreover, Palm et al. provided evidence that, in mice, phospholipase A2 (PLA2), a conserved component of many insect venoms, induced an IL-33-driven T_H_2 response, protecting against subsequent challenge with a lethal dose of PLA2 [130]. This is of particular interest since MCs express the ST2 receptor and are activated strongly by IL-33 [122]. However, MC response to envenomation is not only driven by IgE and IL-33, but they also possess the Mas-related G-protein coupled receptor MRGPRX2 (or its mouse orthologue Mrgprb2) that directly binds venom components, such as wasp venom peptides [131]. Mechanistically, MCs protect from intoxication by directly degrading venoms via proteases or by regulating the immune response [127]. Akahoshi et al. proved that MC chymase is responsible for enhancing resistance to Gila monster venom by directly degrading helodermin, a component of it [132]. Also, protection from two types of scorpion poisons was attributed to MC chymase [132]. Moreover, MC secreted carboxypeptidase A has been shown to protect from snake and bee venoms by a similar mechanism of direct degradation [133]. Importantly, Anderson et al. have recently shown that human MC tryptase can degrade six different snake venoms and have suggested it as a possible therapeutic strategy for treating snakebites [134].

Importantly, although MCs respond to and degrade venoms in general, they can also have a detrimental role. A massive MC reaction to a small amount of poison, such as a single bee sting, may overshoot and lead to anaphylaxis, a complication that may be exacerbated in patients suffering from mast cell activation syndromes (MCAS) [135].

### 2.4. The Role of Mast Cells in Bacterial Infections

**Figure 1:**
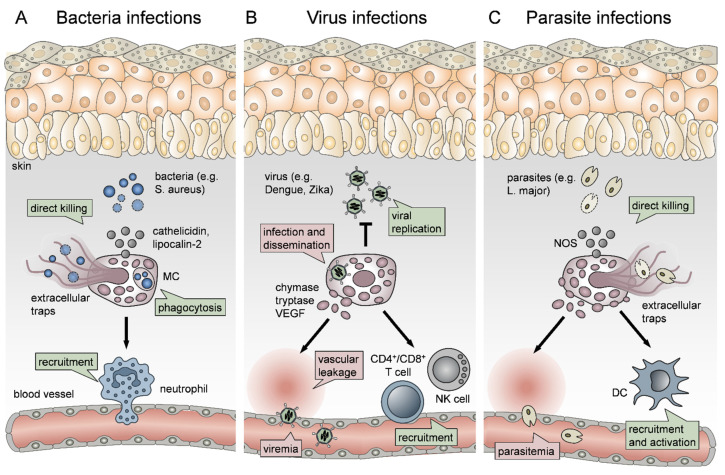
The crucial role and dichotomy of mast cells (MCs) in cutaneous infections. (**A**) MCs critically contribute to host defenses against bacteria via direct killing, phagocytosis and the recruitment of neutrophils. (**B**) In viral infections, MCs control viral replication locally in the skin and promote the recruitment of natural killer cells (NK), CD4^+^ and CD8^+^ T cells, but also contribute to viral dissemination and viremia. (**C**) MCs control parasite infections via the release of nitric oxide species (NOS) and the formation of extracellular traps, as well as by promoting dendritic cell (DC) recruitment and activation, but can also contribute to parasite dissemination. Beneficial MC effects are displayed in green boxes; detrimental MC functions are shown in red boxes.

MCs have been shown to exhibit a protective role in the host defense against a spectrum of bacterial infections [136]. Such infections include *Mycoplasma pneumonia* [137], *Escherichia coli* [138], *Citrobacter rodentium* [139], *Francisella tularensis* [140], *Helicobacter sp.* [141], and *Mycobacterium tuberculosis* [142]. MCs recognize bacteria through TLR signaling and contribute to bacterial clearance by inducing immune cell recruitment and by linking the innate and adaptive immune response through promoting DC maturation (Figure 1A) [83]. For example, Siebenhaar et al. have shown that MC-dependent control of *Pseudomonas aeruginosa* skin infections involves Nph recruitment by the MCs [143]. More recently, Zimmermann et al. delved deeper into *P. aeruginosa* skin wound infection, showing that wound healing was delayed in the absence of MCs. This was attributed to impaired bacterial clearance due to the lack of MC-derived IL-6, which was enhancing the bactericidal properties of KCs [144]. In another case, it was shown that MCs could be activated by co-culture with *Staphylococcus aureus,* a predominantly problematic skin bacterium that can cause serious infections of the skin and lungs and possibly leads to sepsis [145]. Arifuzzaman et al. showed that activation of MCs through the MRGPRX2 receptor, by using the wasp venom component mastoparan, enhanced the clearance of *S. aureus* from infected mouse skin in a process involving Nph recruitment. In addition, MC activation on this occasion led to more pronounced DC migration and stronger protection against re-infection [146].

However, MCs also directly control bacterial infection through phagocytosis, although they are not professional phagocytes, as well as the production of extracellular traps [147] and the release of antimicrobial peptides [148], such as lipocalin 2 and cathelicidin (Figure 1A) [149,150]. For example, Lei et al. have reported that MCs in *S. aureus* infected skin abscess were activated, and MC-derived tryptase was responsible for the inflammation [151]. Also, Nakamura et al. have shown that staphylococcal δ-toxin can activate MCs, leading to degranulation, which may contribute to the exacerbation of atopic dermatitis [152]. However, the release of the antimicrobial peptide with antibiotic properties cathelicidin by skin MCs has been reported to prevent skin infection by invasive *S. aureus* [150]. This is of particular interest when we take into account that IgE mediated mechanisms and MCs are involved in developing acquired immunity against *S. aureus* [153]

Intriguingly, by murine Mrgprb2, or its human ortholog MRGPRX2, MCs have been shown to detect and respond to quorum sensing signals, substances the bacteria use to communicate with each other and coordinate their behavior [154]. In addition, MCs play a significant role in the production of IL-12, which is needed for the protection of the host from polymicrobial infections [155].

Collectively, these reports highlight the important role of MCs in bacterial clearance, particularly in the context of wound healing.

### 2.5. The Role of Mast Cells in Virus Infections

MCs have been implicated with responses against a wide variety of viruses [106,156]. These include respiratory viruses, such as a respiratory syncytial virus (RSV) and parainfluenza, where MCs were associated with infection-driven asthma exacerbation [157,158,159], hepatitis C virus (HCV) [160] and even human immunodeficiency virus (HIV) [161]. Of note, when focusing in this review on MC functions in skin inflammation, MCs critically contribute also to viral infections that take place or start in the skin, such as vectorborne diseases (Figure 1B) [156].

Due to their strategic location and inherent nature as sensors of cell stress and inflammatory insult, MCs respond to inflammation caused by mosquito bite saliva and thus drive plasma leakage, Nph infiltration and draining lymph node (LN) hyperplasia [162]. However, MCs were also reported to downregulate antigen-specific responses to mosquito bites through an IL-10-dependent mechanism [163]. This is extremely important given that the local inflammation and innate response caused by the vector insect bite are often crucial for viral dissemination and infection severity, as shown by Pingen et al. in the context of arbovirus infection [164]. Dengue virus (DENV), which is a mosquito-borne flavivirus, can lead to vascular leakage and hemorrhagic fever, thus causing severe morbidity and mortality [165]. It is widely spread in tropical regions and can affect up to a hundred million people per year. Therefore, understanding its pathogenicity and developing new treatments is of paramount importance [166]. MCs have been shown to be infected by DENV, leading to their degranulation, which can be reduced by DENV neutralizing antibodies [167]. Troupin et al. were the first to report that skin MC infection by DENV is crucial for systemic virus dissemination (Figure 1B) since infectious viral particles localize in secretory granules, which are being trafficked to draining LNs [168]. Moreover, MC collaboration with macrophages (Mph) (Figure 1B) has been reported to control viral replication in the skin [169]. Similarly, MC degranulation-driven recruitment of natural killer (NK) cells and natural killer T cells (NKT) has been shown to promote viral clearance in the mouse model (Figure 1B) [170].

However, MCs are not only implicated with DENV dissemination and local skin clearance but also pathology since the innate response to the virus is underlying disease exacerbation [165]. A clinical study by Furuta et al. has shown that MC-derived VEGF, tryptase and chymase contribute to DENV shock syndrome [171]. Another clinical study reported that antibody-mediated MC activation leads to vascular leakage (Figure 1B) during DENV infection [100]. This is further supported by the fact that a high chymase serum level is a prognostic factor for DENV hemorrhagic fever [172]. Given that MCs mainly promote DENV infection, MCs have been proposed as potential therapeutic targets [173]. However, a recent study by Mantri et al. has reported a beneficial role for MCs in DENV infection. More specifically, MCs were shown to form immunological synapses with γδ T cells, leading to their activation and killing of infected DCs, which resulted in controlling the virus [115].

As another flavivirus, the Zika virus has been recognized as a threat to international health after the 2016 outbreak. Zika is a mosquito-borne virus that can lead to congenital defects when passed from the mother to the embryo through the placenta [174]. Similar to DENV, human placental MCs and a human MC cell line have been recently reported to be infected by Zika [175]. In addition, in an in vitro study, MCs have been associated with contributing to Zika virus pathology because MCs from a human cell line have been shown to be infected by the Zika virus and produce viral particles [176].

### 2.6. The Role of Mast Cells in Parasite Infections

MCs are implicated in cutaneous parasite infections, and, similarly to vectorborne viruses, in cases where parasites are disseminated by insect bites through the skin, such as infection with *Leishmania* sp., *Plasmodium* sp. and *Trypanosoma* sp. [177]. For example, in the case of malaria, when mice were infected with *Plasmodium berghei,* MCs were observed near sporozoites of the parasite at the site of the mosquito bite [178]. Moreover, a clinical study by Wilainam et al. showed that MC degranulation in the skin of patients with *Plasmodium falciparum* infection was a strong indicator of parasitemia (Figure 1C) and disease severity [179]. Another study has supported the notion that MCs promote *Plasmodium* sp. dissemination by showing that *Plasmodium berghei* infection of mice caused massive MC degranulation in the skin and draining LNs. In this context, disease severity was increased when mice were treated with the MC activator c48/80, while the disease was ameliorated upon treatment with the MC stabilizer DSCG [180].

In addition to vectorborne parasites, we herein focus on *Leishmania* sp. since they cause a predominantly cutaneous infection that can progress into more systemic forms, such as visceral leishmaniasis [181]. Leishmania promastigotes mainly infect Mph, but *L. major* and *L. infantum* have been reported to infect MCs directly [182]. For canine leishmaniosis, as well as human infection with *Leishmania brasiliensis*, MC accumulation at the site of infection has been reported [183,184]. Moreover, the number of MCs at the site of infection was associated with clinical disease progression [183]. MC numbers further seem to correlate with parasite infection since *L. major* susceptible BALB/c mice showed elevated MC numbers at the lesion site, in contrast to resistant C57BL/6 mice [181]. Furthermore, MCs seem to be crucial for controlling the infection since MC-deficient mice develop larger lesions with higher parasite loads and are prone to frequent dissemination of *L. major* to the spleen. In the absence of MCs, fewer DCs are recruited to the lesion (Figure 1C), leading to a deficit of IL-12, which is necessary for developing a healing T_H_1 response [185]. Another important study has shown that MCs can not only recruit DCs to the site of *L. major* infection but also directly interact with them (Figure 1C). Subsequent T cell activation by DCs that underwent this crosstalk led to the induction of a T_H_1 phenotype [14]. However, more recent evidence shows that MCs do not only have a protective role against *Leishmania* sp. infection through activating DCs but can directly kill the parasites by the production of NOS and the formation of extracellular traps (Figure 1C) [186]. Interestingly, Paul et al. reported in 2016 that MCs are dispensable for cutaneous leishmaniosis, introducing controversy on the topic [187]. Nevertheless, most published reports suggest a rather beneficial role of MCs in *L. major* infection.

### 2.7. The Role of Mast Cells in Fungi Infections

Fungi comprise a group of eukaryotic organisms, members of which cause serious infections, affecting approximately 300 million people a year. These infections are often initiated from environment-barrier interfaces, such as the skin and the lung mucosa [188]. MCs may contribute to host defense against fungi since they populate these interfaces in high densities and can sense pathogen products, for example, through TLR or C-type Lectin receptor signaling [89,156]. However, most studies have focused on Mph, monocytes (Mo) and Nph, as well as ECs, rather than on direct MC antifungal activity and orchestration of the immune response [189,190,191].

Nevertheless, MCs have been reported to play a crucial role in various fungal infections [188]. In the case of *C. albicans,* a fungus that can infect the skin but can also cause systemic infections, a recent study has shown that recognition of the fungi by rat bone marrow-derived MCs (BMMCs) through the C lectin type receptor (CLR) Dectin 1 led to MC degranulation and release of TNF-α, IL-6, IL-10, CCL2, CCL4 and NOS [192]. Moreover, Lopes et al. reported that, in vitro, human MCs could mount specific early, mid and late responses against *C. albicans.* More specifically, MCs were able to phagocytose the fungi and reduce its viability, followed by recruitment of Nph. In addition, infected MCs formed extracellular DNA traps suggesting, overall, a protective role of MCs against *Candida* infection [193]. Indeed, MCs were reported in vivo to kill extracellular but not ingested *C. albicans* in a process potentially involving degranulation [194].

However, MCs do not always have a protective role in fungi infections. In lung infections with *Aspergillus fumigatus*, resulting in IgE-mediated allergic bronchopulmonary aspergillosis (ABPA), MC proteases are responsible for releasing growth factors from ECs, promoting lesions and fibrosis [195]. This is further supported by the fact that omalizumab, a monoclonal antibody against IgE, is an effective treatment for ABPA [196], but *A. fumigatus* can also activate MCs in an IgE independent manner [197]. Moreover, extracts of *Malassezia sporodialis*, a species usually associated with cutaneous diseases and considered to contribute to developing atopic dermatitis [198], have been proven to cause MC activation. Mechanistically, binding of the CLR Dectin 1 to the fungi component curdlan leads to MC degranulation and release of Leukotriene C_4_, IL-6 and CCL2 [199]. In vitro, Ribbing et al. have also shown that human MCs can detect and respond to *Malassezia* sp. by engagement of the CLRs Dectin 1 and Mincle [200].

## 3. Mast Cell Contribution to Inflammatory Skin Disorders

### 3.1. IgE-Mediated Acute Allergic Cutaneous Responses

MCs are well-known as key effector cells in type I allergic responses. As described above, MCs respond to IgE/FcεRI crosslinking by immediate degranulation of secretory granules. Intriguingly, perivascular MCs can sample IgE from the bloodstream by generating processes through the vessel wall [201]. Granule-bound histamine, which becomes soluble almost immediately, is a key driver of vasodilatation and increased vessel permeability, ultimately resulting in rapid local edema formation. Granule-embedded cytokines and chemokines, including TNF, IL-6, CXCL-1 and eotaxins, account for subsequent effector cell recruitment, including Nph and eosinophil granulocytes (Eos) and Mo [16]. The orchestration of cell recruitment by MCs is further amplified by subsequent de novo synthesis of lipid mediators and additional cytokines and chemokines. This, consequently, manifests clinically as locally restricted early edema, compared to more disseminated and hardened skin edema at late time points. Importantly, due to the plethora of released mediators and multiple overlapping pathways, even local allergen encounters and MC activation may culminate in life-threatening systemic anaphylaxis. The severity of anaphylaxis was shown to increase with high plasma heparin levels resulting in factor XII autoactivation and bradykinin formation [202]. Importantly, the mechanisms converting a local reaction into a systemic anaphylactic response and defining the severity of an anaphylactic reaction remain unknown [203].

### 3.2. The Role of Mast Cells in Atopic Dermatitis

**Figure 2:**
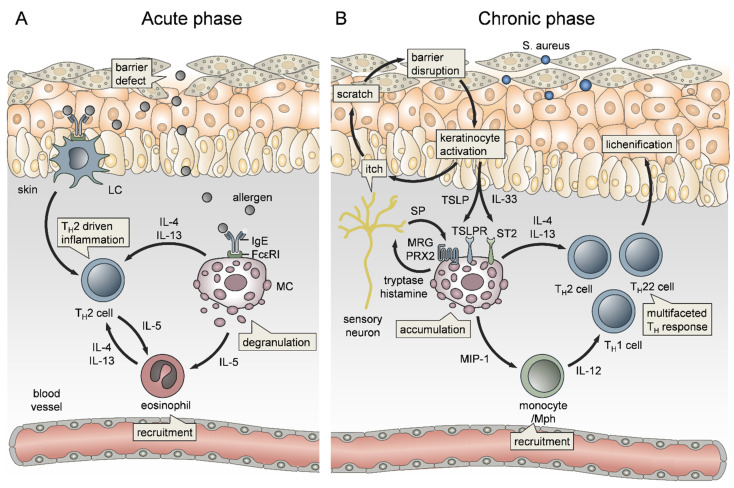
The role of mast cells (MCs) in the vicious cycle of atopic dermatitis. (**A**) In the acute phase of atopic dermatitis (AD), allergens crosslink IgE antibodies bound to FcεRI on Langerhans cells (LC) and MCs. MC degranulation and cytokine release induce T_H_2 cell and eosinophil recruitment, thereby leading to a self-perpetuating cycle of T_H_2-driven inflammation. (**B**) The chronic phase of AD is characterized by a multifaceted T_H_ response, which is perpetuated by an IgE-independent MC–nerve-skin axis. Itch and scratching promote keratinocyte (KC) activation and release of thymic stromal lymphopoietin (TSLP) and IL-33, thereby activating MCs through the TSLP receptor (TSPLR) and ST2 receptor, respectively. TSLP can also promote MRGPRX2 signaling. MC release of tryptase and histamine activates neurons, thus promoting itch and, in turn, activating MCs by neuropeptides, such as substance P (SP), through the Mas-related G-protein coupled receptor X2 (MRGPRX2). MC-derived macrophage inflammatory protein 1 (MIP-1) recruits monocytes and macrophages (Mph), which together with MCs promote T cell inflammation, finally leading to skin lichenification.

Atopic diseases are a family of IgE-mediated type I hypersensitivities, including atopic dermatitis (AD), allergic rhinitis, allergic asthma, food allergy and life-threatening anaphylaxis. AD is a chronic recurrent inflammatory skin disease affecting about 30% of children and is characterized by a T_H_2-cell dominated immune response, itching and impaired skin barrier (Figure 2A) [204]. Importantly, children with an early onset and persistent disease have a high risk of developing allergic asthma, a phenomenon known as “atopic march” [203,205]. AD comprises several endotypes between age groups and ethnicities, characterized by IgE levels and filaggrin mutation status, making traditional non-targeted therapies tricky [206]. Despite the elevated IgE serum levels, the role of MCs in chronic AD is still incompletely understood. Given the expression of FcεRI on Langerhans cells and Mph in chronic AD, an important role for IgE in allergen presentation and expansion of allergen-specific IL-4/IL-5-producing T_H_2 cells, promoting the subsequent infiltration of Eos (Figure 2A), has been proposed [207,208,209]. However, MCs accumulate in chronic AD lesions and even migrate into the epidermis [210]. Since most papillary and epidermal MCs were localized close to ECs, they may be involved in neoangiogenesis by expressing proangiogenic factors [211]. In addition, MCs have been demonstrated as a source of IL-4 [46], IL-5 [209] and IL-13 [212], thereby likely involved in the IgE/T_H_2/Eos vicious cycle (Figure 2). Importantly, IL-4 induced MC expansion and functionally re-shaped human skin MCs towards increased FcεRI expression and boosted histamine synthesis and release [213] while, in turn, repeated FcεRI triggering modified the MC transcriptome [214]. In line with this, even progenitor-derived MCs from AD patients differ from healthy controls by having enhanced levels of granule mediators and IL-6 responsiveness, indicating a link to the genetic predisposition of atopic disorders [200].

Depending on the route of activation, MCs can bridge innate and adaptive immunity and thereby contribute to allergic sensitization in the skin and lung [215]. For example, MC-derived IL-13 has been demonstrated to downregulate IL-12 production by skin DCs, thereby inhibiting the T_H_1 cell response to cutaneous antigen exposure [216]. Likewise, MCs counter-regulate IFN-γ expression in sensitized skin [217]. The pattern of cytokine expression in AD depends on the acuity or duration of the skin lesion. The acute onset of skin inflammation is associated with a predominance of T_H_2 cell infiltration and IL-4 expression (Figure 2A). In contrast, macrophage and Eos activation are dominant in chronic AD, where MC-derived cytokines, chemokines and MC interaction with the vessel endothelium contribute to monocyte recruitment (Figure 2B) [218,219]. The ongoing cytokine expression, local expansion of Th2 cells and pathologic keratinocyte damage caused by scratching or microbial agents (e.g., *Staphylococcus aureus*) amplify tissue inflammation. KCs release a spectrum of cytokines, such as IL-1β, IL-25, IL-33, thymic stromal lymphopoietin (TSLP), as well as the alarmins ATP and HMGB1 that skew DC towards type 2 immunity, further boosting MC effector functions [220]. Specifically, in AD, TLSP stimulates MC accumulation and Th2 cytokine production, which directs epithelial cell-mediated, IgE-independent MC activation that exacerbates disease severity (Figure 2B) [221,222,223,224]. Furthermore, it has been recently shown that TSLP can activate human MCs directly through cooperating with MRGPRX2, shedding some light on its IgE-independent mode of action [225]. Notably, TSLP is also produced by MCs in a caspase-1/NFκB-dependent way [226]. In line with TSLP, IL-33 release by KCs acts on T_H_2 cells, MCs and Eos, the key drivers of AD (Figure 2B), via the specific receptor ST2 [227]. IL-33 affects several MC functions, including growth, survival, and mediator release (as reviewed in [228]). In vitro, IL-33 was thought to fail direct induction of MC degranulation but to amplify the release of de novo synthesized lipid mediators and cytokines. However, we demonstrated that IL-33 initiates MC degranulation and MC-mediated edema formation and Nph recruitment in vivo [90,229], which may result from concomitant signaling of extracellular ATP via the purinergic receptor P2X7. Indeed, transgenic expression of IL-33 by KCs resulted in MC accumulation, increased blood histamine and total IgE levels, and increased levels of IL-5, IL-13, CCL5, and eotaxin 1 in blood and lesional skin, thereby closely resembling the AD features [230]. Notably, in a murine model, MCs were found to express MHC class II after prolonged exposure to IL-33, suggesting a possible role for MCs in promoting the vicious cycle of response to/induction of type 2 immunity [231].

AD exacerbation via IgE-independent MC activation is also triggered by the Mas-related G-protein coupled receptor MRGPRX2 (or its murine orthologue Mrgprb2) (Figure 2B). For example, as a model for AD, imiquimod application induced dermatitis with inflammatory cell infiltrates MC activation and increased histamine and cytokine serum levels in wt mice, but not in MRGPRB2^−/−^ mice [232]. In addition, antimicrobial peptides, neuropeptides, major basic protein, eosinophil peroxidase, and many FDA-approved peptidergic drugs activate human MRGPRX2 and may result in pseudo-allergic responses [233,234]. Recent work on MRGPRX2-mediated signaling of neuropeptides in MCs demonstrated MC/nerve communication (Figure 2B), which is amplified in AD due to MC accumulation, MC/nerve-connections and increased Substance P levels (reviewed in detail in [235,236,237]. Serhan et al. demonstrated that house dust mite (HDM)-activated skin peptidergic nociceptors drive type 2 skin inflammation by induction of MC degranulation through the release of substance P [238]. Thus, MC mediators play a key role in linking nociception to skin inflammation and pruritus [239,240]. In AD, itch, one of the most burdensome hallmarks, is triggered by histaminergic routes [241,242,243] but also by non-histaminergic, but tryptase-dependent, mechanisms (Figure 2B), including MRGPRX2 activation [233,244,245,246,247]. Importantly, itch and scratching facilitate the encounter of external triggers, such as *S. aureus*, partially activating MRGPRX2 themselves and consequently further promoting the vicious cycle towards disease exacerbation (Figure 2B). For example, *S. aureus* δ-toxin and *S. aureus* enterotoxin B (SEB) enhance allergic skin inflammation by activating MCs, the latter via increased expression of IL-33 and ST2 [152,248,249].

Furthermore, pruritus as a main characteristic of AD explains the link between AD and food allergies. Leyva-Castillo et al. showed recently that mechanical skin injury causes expansion and activation of intestinal MCs, increases intestinal permeability and thereby promotes food anaphylaxis in sensitized mice [250]. Notably, the remote association between AD and food allergies again includes IL-33 and TLSP effects on MC degranulation [251].

Recent knowledge may be helpful for identifying potential biomarkers for disease progression and targets for therapeutic strategies, particularly to intervene in the atopic march. Indeed, there is already considerable effort to not only improve the treatment of histaminergic effects [252] but also to include biological therapies targeting the type 2 immune response, involving Th2 cells, MCs and others, such as IL-4, IL-5, IL-13, IL-31, TNF, TLSP [253,254] and IL-33 [255].

### 3.3. Mast Cell Functions in Contact Hypersensitivity

**Figure 3:**
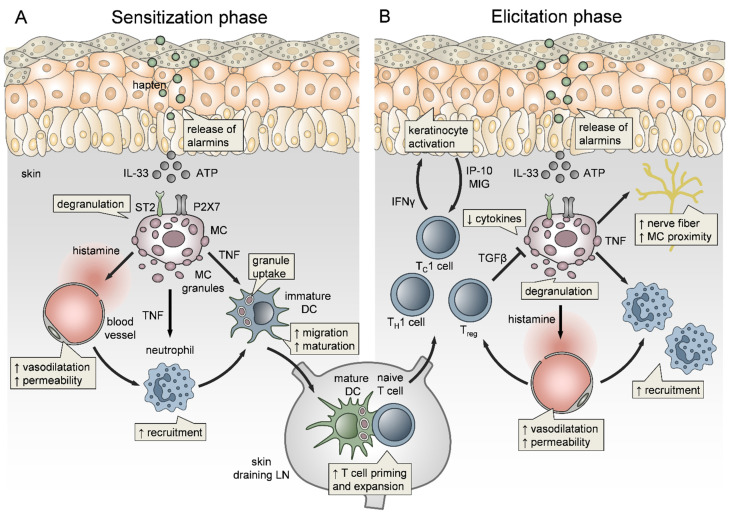
Mast cell (MC) functions in allergic contact dermatitis. (**A**) In the sensitization phase of allergic contact dermatitis (ACD), or its mouse model of contact hypersensitivity (CHS), MCs sense keratinocyte (KC) cell stress due to their release of alarmins, such as IL-33 and ATP. Then, concomitant IL-33/ST2 and ATP/P2X7 signaling lead to the activation and degranulation of MCs. While vasodilation and vascular permeability are driven by histamine, MC-derived TNF primes and recruits neutrophils. Moreover, MC-derived TNF and MC granules promote dendritic cell (DC) maturation and migration to the skin draining lymph node (LN), thereby enhancing T cell priming. (**B**) Upon reexposure, MCs once more initiate vascular responses and neutrophil recruitment, but also nerve fiber elongation. Additionally, skin inflammation is amplified by infiltration of T_H_1 and cytotoxic T cells (T_C_1). Interferon γ (IFNγ) released by TC1 cells activates KCs, which in turn enhances the skin inflammation in a feedback loop by the production of the T cell recruiting chemokines Interferon γ-induced protein 10 (IP-10) and monokine-induced by gamma interferon (MIG). MC cytokine production is counter-regulated by regulatory T cells (T_reg_) via the release of transforming growth factor β (TGFβ).

Affecting about 11% of women and 5% of men in industrialized countries, allergic contact dermatitis (ACD) is one of the most common occupational diseases [256,257,258]. ACD is a chronic skin inflammatory disorder driven by a T cell-mediated delayed-type hypersensitivity (DTH) response (Figure 3) to low-molecular-weight organic chemicals or metal ions (reviewed in [259,260]). During sensitization, these compounds, referred to as “haptens”, penetrate into the skin and bind to self-proteins in the dermis, thereby rendering them antigenic. Skin resident DCs take up the “haptenized” proteins and prime allergen-specific T cells in skin-draining LNs (Figure 3). Upon every following hapten encounter, referred to as “elicitation”, hapten-specific T cells infiltrate the allergen-exposed skin and mediate a Th1, and CD8^+^ T cell dominated skin inflammatory response [261].

The immune events underlying ACD have been widely studied with the help of experimental mouse models for contact sensitization (contact hypersensitivity, CHS), which is the DTH response to small sensitizing organic haptens, such as DNFB or TNCB [262]. Research of the last decades revealed that an innate immune response involving pattern recognition receptor-mediated sensing and infiltration and effector function of various immune cell types precedes the T cell-mediated inflammation [263,264]. Potently sensitizing haptens evoke a local inflammatory response within the first hours after administration (Figure 3A), providing adjuvant effects that promote the induction of the subsequent allergen-specific T cell response [265,266].

In the early 1990s, MCs have been suspected of promoting DTH elicitation by vasoactive mediators. However, the analysis of CHS responses in *Kit* mutant mice, as a model for MC deficiency, revealed controversial data ranging from defective [267], normal [268,269] to even enhanced [269] skin inflammation. However, as early as 1987, very early onset of MC degranulation, ear swelling and serum histamine levels in the first hours after hapten treatment have been reported [270]. Demonstrating that MC functions in sensing and initiation of innate responses can be easily overlooked by analyzing late time points, which in CHS are classically 24 h or 48 h after hapten challenge. Nevertheless, there is increasing evidence not only of critical MC relevance in skin inflammation upon hapten elicitation but also of contribution to sensitization (Figure 3A) (reviewed in [258,262,263,271]). Using a novel transgenic mouse model of MC deficiency, independent of *kit* mutations, we have demonstrated that MCs are key promoters of CHS and mediate the early adjuvant effects of several haptens [94]. Importantly, MC deficiency resulted in diminished edema formation and Nph and T cell skin infiltration upon elicitation, but also impaired migration of skin DCs to the LNs and reduced T cell priming upon hapten sensitization. Given the rapid MC response, we questioned the mode of activation. We found that MCs sense cell stress and tissue damage via concomitant IL-33/ST2 and ATP/P2X7 signaling, while MyD88-dependent TLR signaling is not involved [228]. Efficient MC activation in CHS responses also depends on FcεRI α-chain/ITAM signaling [272], is enhanced by antigen-independent effects of IgE [273], and may include inflammasome activation [274]. Hence, epidermal responses to hapten encounter, e.g., by keratinocytes and dendritic epidermal T cells (DETC) [259,264,275], seem to precede MC activation. In vitro, the formation and internalization of hapten–protein complexes by human keratinocytes has been shown to result in neoepitope formation [276,277,278]. While in vivo, the sequence of events is less understood, epidermal stress responses include inflammasome activation and release of IL-1β and IL-18 [279,280], production of reactive oxygen species (ROS) [281] and alarmins, e.g., ATP [282,283] and IL-33 [284,285,286,287]. The subsequent MC activation rapidly elicits skin blood vessel vasodilatation and increased vessel permeability, resulting in biphasic edema formation, peaking on the first 2 h after hapten encounter and followed by a second peak after 24 to 48 h in sensitized mice, that is predominantly driven by histamine effects [228]. Histamine has also been shown to impact sensitization to nickel [274] and to contribute to chronic ACD [288]. In addition to histamine-driven dysregulation of endothelial barrier integrity, we and others demonstrated a crucial role of MCs, particularly of MC-derived TNF (Figure 3), in the initiation of Nph infiltration to hapten-challenged ear skin [70,94,289]. Biedermann and colleagues demonstrated that MCs promote Nph recruitment by the release of MIP-2 and TNF [70], where, mechanistically, TNF may directly impact TNFR1-expressing ECs [71]. In a recent report, we could show in a mouse model of conditional TNF inactivation in CTMCs, that MC-derived TNF is dispensable for the induction of endothelial cell adhesion molecules. In contrast, we determined that perivascular MCs have the capacity to pass the vessel wall and directionally degranulate into the vessel lumen. After being infused into the bloodstream, MC-TNF directly primes circulating Nph via TNFR1 on the Nph surface and thereby allows their efficient extravasation to the inflamed skin [15]. Importantly, MC-mediated early recruitment of Nph is not only the driving force behind the amplitude of skin inflammation upon elicitation but is also required for efficient sensitization. Weber et al. showed a crucial role for Nph in DC migration to skin draining LNs and priming of allergen-specific T cells [288]. Besides controlling skin Nph numbers, MCs have been shown to contribute to hapten sensitization in various modes of action. Wang et al. reported that upon hapten sensitization, MCs degranulate rapidly, within 30 min, in the affected skin but later on accumulate in the skin draining LNs where they may contribute to T cell priming [290]. Moreover, a direct interaction between MCs and DCs in the skin upon hapten sensitization has been demonstrated by analyzing skin biopsies [291], as well as by dynamic in vivo analysis using intravital multiphoton microscopy [112]. Importantly, in vitro and in vivo data indicate an important role for MCs, and MC-derived TNF, in promoting the maturation of DCs and their subsequent migration to skin draining LNs (Figure 3A) [14,290,292]. Of note, MC-derived TNF amplifies predominantly the migration and function of the cDC1 subtype (e.g., CD103^+^ skin DCs reflecting the CD8^+^ LN DCs) and thereby the priming and expansion of CD8^+^ T cells [293], which have been previously shown to be dominant effector cells in hapten-induced skin inflammation [259]. This specification may be related to the mechanistic impact of MC secretory granules. We could show, that both upon LPS-induced and hapten-driven MC degranulation, the exocytosed intact granules are actively engulfed by neighboring DCs and shuttled to skin draining LNs [106]. Importantly, this MC granule uptake accelerated DC migration, facilitated DC maturation and therefore, boosted T cell priming (Figure 3A). Given the fact, that CD103^+^ skin cDC1 were most efficient in MC granule uptake, but that this advantage was undone in the absence of MC-TNF, MC-TNF effects on cDC1 functions may occur due to enhanced MC granule uptake. Most importantly, the impact of MCs on DC functionality is simultaneously mirrored by a mutual influence of DCs on MC functions. The hapten-induced dynamic interaction between MCs and DCs observed by intravital imaging, culminated in innate synapses and MHCII transfer from DCs to MCs. Consequently, MCs acquired antigen presenting capacity, which may contribute to effector T cell activation and T cell-mediated skin inflammation upon hapten elicitation [112].

Notably, MC-derived TNF has been additionally shown to promote nerve fiber elongation during CHS, as well as closer MC proximity to nerves (Figure 3B) [294]. Since ACD is manifested as a pruritic inflammatory skin disorder, MC’s contribution to both histaminergic and non-histaminergic itch might be considered [295]. Moreover, models of repeated antigen challenge have been used to study MC effects in chronic ACD. Here, MCs accumulate in an L-selectin and ICAM-1 dependent manner and shift the delayed-type to an immediate-type response [296]. Interestingly, Gimenez-Rivera demonstrated that MCs limit the exacerbation of chronic ACD by controlling T_H_1 and T_H_17 cytokines, and CD8^+^ tissue-resident memory T (T_RM_) cells, probably by degradation of the CD8^+^ T cell mitogen IL-15 [297]. In vitro, persistent exposure of human MCs to IL-33, as occurring in chronic inflammatory skin disorders, attenuated degranulation and FcεRI expression but induced amplified histamine production [298].

Of note, NF-κB-induced proinflammatory cytokine production by MCs, upon CHS, is counter-regulated by iT_reg_ cells via TGF-β (Figure 3B) [299]. Moreover, MCs seem to elicit not only proinflammatory but also immunoregulatory functions in ACD (reviewed in [300,301,302]). While in low-dose CHS responses, MCs promote the early onset and magnitude of skin inflammation, MCs may regulate more severe CHS responses and cytokine patterns upon high-dose hapten challenge [303,304]. Mechanistically, MCs suppress severe CHS by the release of IL-10 [245,305], by maintaining IL-10 producing regulatory B cells, through the secretion of IL-5 [306], and by the PD-L1-mediated suppression of CD8^+^ effector T cell activation [307].

### 3.4. Mast Cells in Psoriasis

**Figure 4:**
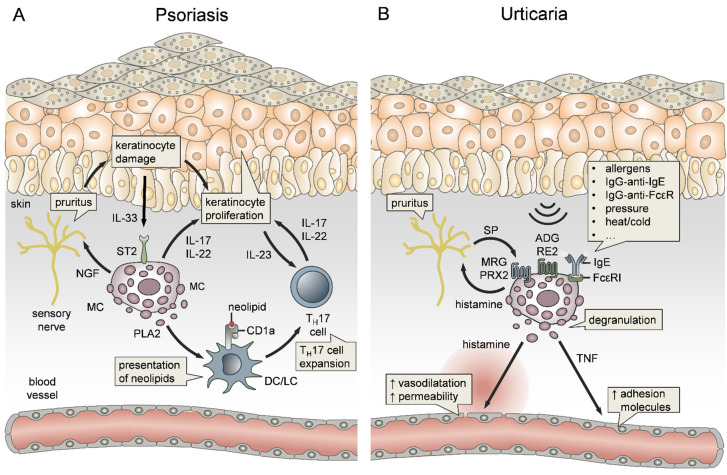
The role of mast cells (MCs) in psoriasis and urticaria. (**A**) MCs directly contribute to psoriatic plaque formation via the release of the keratinocyte (KC) proliferation-inducing cytokines IL-17 and IL-22. Indirectly, MCs promote T_H_17 cell expansion via the production of phospholipase A2 (PLA2), which is taken up by dendritic cells (DC) and Langerhans cells (LC), leading to the presentation of neolipids through CD1a. Additionally, MCs promote pruritus through the release of nerve growth factor (NGF) that causes KC damage. Consequently, IL-33 production by KCs activates MCs through the ST2 receptor, thus ending in a self-perpetuating cycle. (**B**) In urticaria, MC activation and degranulation can be induced by numerous stimuli, including autoimmune and mechanical triggers. While histamine promotes vasodilation and vascular permeability, MC-derived TNF induces the expression of endothelial cell adhesion molecules. Moreover, tryptase and histamine are activating neurons, leading to itching and release of neuropeptides, such as substance P (SP). SP, in turn, activates MCs in an IgE-independent manner via the Mas-related G-protein coupled receptor X2 (MRGPRX2). Another IgE-independent mechanism of MC activation is through the mechanical sensing receptor ADGRE2.

Psoriasis is considered to be an autoimmune disorder that is characterized by massive immune cell infiltration into the dermis and epidermis, followed by an abnormal proliferation of KCs [308]. The skin disease is perpetuated by both innate and adaptive mechanisms (Figure 4A) and, although the inflammation appears locally in the skin, it involves detrimental effects on patient quality of life and may be even accompanied by systemic manifestations [309]. MCs have been investigated early in the context of psoriasis, and it was demonstrated that MC degranulation is a constant and early feature in disease development [310]. Another study showed that MCs in psoriatic skin lesions were functionally hyperactive and suspected that histamine is involved in disease pathophysiology [311]. Indeed, MCs have been implicated in the onset of neuropathic pain, itching and pruritus (Figure 4A), the latter being some of the main symptoms of psoriasis [245]. This is triggered by a plethora of MC mediators, including histamine, tryptase, cytokines and growth factors [240]. In a recent study, Nobuo et al. reported the relationship between MCs and pruritus in a mouse model of imiquimod-induced psoriasis, showing that self-scratching behavior during the onset of psoriasis led to increased MC numbers, which, in turn, induce pruritus through the release of nerve growth factor (NGF) (Figure 4A) [312]. Notably, a recent study has employed transcriptional profiling to establish a common signature for itchy psoriatic and AD skin, showing that many MC genes, such as tryptase, phospholipase A2 (PLA2), IL-6, IL-17, IL-22 and MRGPRX2, were commonly upregulated between the two types of pruritus [313].

In addition to the onset of pruritus, MCs play an active role in driving disease pathogenesis [314]. Mechanistically, IL-33 levels were found to be increased in human psoriatic skin (Figure 4A), and, by stimulating MCs to release VEGF, contributed to the inflammation [315]. This is also supported by evidence that IL-33 in psoriatic plaques is secreted by KCs in response to inflammatory stimuli [316]. Most importantly, MCs have been shown to be major producers of IL-17 and IL-22 in human psoriatic skin, which drive the disease by inducing uncontrolled keratinocyte proliferation and psoriatic plaque formation (Figure 4A), suggesting MC-driven disease exacerbation [317,318]. On this note, a study by Lun et al. reported that PLA2, released by MCs through degranulation, is taken up by CD1a-expressing antigen-presenting cells in psoriatic skin. This promotes the generation of neolipid antigens, which are specifically recognized by CD1a-reactive T cells, leading to the release of IL-17 and IL-22 [123]. Therefore, MCs are not only driving the disease by direct production of IL-17 and IL-22 but also indirectly through the perpetuation of T cell activation (Figure 4A). Considering that MCs promote both the pathomechanism of psoriasis and psoriasis-associated pruritus, MCs represent potential therapeutic targets.

## 4. Mast Cell-Driven Skin Diseases

### 4.1. The Role of Mast Cells in Urticaria

Urticaria is a common skin disease that is characterized by transient erythematous swelling of the skin. It can be categorized into acute and chronic urticaria, or according to the clinical course of the disease, into spontaneous, physical (inducible) and other types of urticaria [319]. MCs are key effector cells in the pathogenesis of urticaria, mainly via the release of high amounts of the vasoactive mediator histamine (Figure 4B). Both MC degranulation in lesional skin and increased plasma concentrations of histamine, were observed in patients with chronic spontaneous urticaria (CSU) [320,321], symptomatic dermographism [322,323], cold-induced urticaria [324,325], heat-induced urticaria [326,327], solar urticaria [328], cholinergic urticaria [325,329] and delayed pressure urticaria [322,330]. MC-derived histamine promotes vasodilatation and a local increase in vascular permeability, leading to the prominent symptoms of urticaria, such as wheal formation and angioedema (Figure 4B). Therefore, histamine receptor antagonists, antihistamines, are used as an often inefficient first-line therapy in acute and chronic urticaria [331]. Besides histamine, MC-derived TNF was shown to play an important role in the pathogenesis of CSU by inducing the expression of EC adhesion molecules, including ICAM-1, VCAM-1 and E-Selectin, consequently promoting the recruitment of other immune cells to lesional skin (Figure 4B) [236]. The triggers of MC activation and subsequent degranulation in urticaria are heterogeneous and still not completely understood. In CSU, IgE antibodies against local autoallergens (type I autoimmunity), as well as IgG autoantibodies against IgE or the high-affinity IgE receptor FcεRI (type II autoimmunity) are considered to be the main causes for MC degranulation (Figure 4B) [332]. Based on this, the anti-IgE antibody Omalizumab is an effective therapeutic tool in severe CSU, especially for patients in whom antihistamine treatment is insufficient [333]. Intriguingly, omalizumab also reduced symptoms in patients with symptomatic dermographism, cold urticaria and solar urticaria, indicating a role for IgE-dependent pathomechanisms in physical urticaria [334]. However, recent studies have also revealed IgE-independent mechanisms of MC activation in physical urticaria. Boyden et al. could show, that patients with vibratory urticaria carry a specific mutation in the auto inhibitory subunit of the ADGRE2 receptor, causing hyperresponsiveness of MCs to vibratory stimuli [335]. This finding highlights the involvement of the ADGRE2 receptor in mechanical sensing by MCs. Apart from this, the neuropeptide substance P (SP) might play a key role in the pathogenesis of urticaria (Figure 4B). SP was shown to be increased in the skin of urticaria patients and to induce wheal and flare formation [336,337]. In this line, Fujisawa et al. could recently show, that MRGPRX2, which recognizes SP, is upregulated in skin MCs of CSU patients. Moreover, SP, as well as the Eos-derived mediators major basic protein (MBP) and eosinophil peroxidase, induced the MRGPRX2-dependent release of histamine by human skin MCs (Figure 4B) [338]. Based on this the authors concluded, that MRGPRX2 may be a potential target for therapeutic strategies aimed at alleviating urticaria.

### 4.2. Mast Cell-Driven Mechanisms in Mastocytosis

Mastocytosis represents a heterogeneous group of primary MC disorders, which result from a clonal expansion and accumulation of MCs in multiple organs (systemic mastocytosis, SM) or predominantly in the skin (cutaneous mastocytosis, CM). The main causes of mastocytosis are hypermorphic mutations in the proto-oncogene *KIT* (also referred to as c-kit). Whereas the *KIT* D816V mutation is present in nearly all SM cases (>80%) and can be targeted for therapy [339], the mutation pattern in CM patients varies [340]. *KIT* encodes for the receptor tyrosine kinase c-kit (CD117), which is the receptor for the MC growth factor SCF [341]. Consequently, c-kit constitutively induces MC proliferation and promotes MC survival mainly via the upregulation of anti-apoptotic molecules [341]. While MCs in the bone marrow of mastocytosis patients show enhanced expression of MCL-1 and Bcl-xL, cutaneous MCs exclusively upregulate Bcl-2, suggesting differential regulation of MC survival in this disease [342,343]. CM can be categorized, according to the characteristics and distribution of skin lesions, into maculopapular CM (also referred to as urticaria pigmentosa), diffuse CM and mastocytoma of the skin [344]. The lesions in CM typically consist of red-brown, itchy macules of varying sizes, which result from local MC accumulation and consequent MC degranulation. Therefore, oral antihistamines and topical corticosteroid treatment are used as first-line therapy to alleviate symptoms [345]. Moreover, tyrosine kinase inhibitors, including imatinib and midostaurin, are now approved for the treatment of systemic mastocytosis and may also be promising for the therapy of CM [346]. However, until now, no cure for mastocytosis has been found.

### 4.3. Mast Cell Activation Syndrome

Mast cell activation syndrome (MCAS) encompasses the second group of primary MC disorders, which result from chronic aberrant constitutive and reactive MC activation, without MC neoplasia, as observed in mastocytosis. While *KIT*-mutation-based clonal MC expansion is referred to as a primary MC disorder, MCAS can be further categorized as (1) secondary MCAS with an underlying IgE-dependent allergy or other reactive MCA-triggering pathology; or (2) idiopathic MCAS, where neither a triggering reactive state nor *KIT*-mutated MCs is identified [347]. While the exact pathomechanism is still unknown, Molderings et al. could show a familial occurrence of MCAS, suggesting a genetic component in this disease [348]. MCAS patients show an extreme heterogeneity of clinical symptoms, including dermatologic, lymphatic, pulmonary, cardiovascular, gastrointestinal, musculoskeletal, neurologic or constitutional disorders, which complicates the exact diagnosis [349]. Skin symptoms of MCAS, including itching, wheeling and flushing, are predominantly caused by the release of histamine and prostaglandin D2 [350]. Therefore, H1 antihistamines and MC-stabilizing agents, such as cromolyn sodium, are the first-line therapy in MCAS [351].

## 5. Conclusions

MCs are equipped with a plethora of receptors to sense invading pathogens or cell stress and tissue damage. In addition, skin MCs are strategically positioned beneath the epidermal barrier and attached to the endothelial barrier to translating danger signals into systemic signals, recruiting further immune effector cells. This communication axis is mediated by various mediators, including histamine, cytokines, chemokines, growth factors and proteases. The rapid response by degranulation, their capacity to degranulate into the bloodstream and the huge amount of preformed mediators make MCs be of crucial importance for the first-line defense against pathogens. However, when overshooting, the same features are responsible for detrimental effects in pathogen dissemination and disease exacerbation. Moreover, MCs are not only responsible for skin inflammation in the classic MC-driven skin disorders, such as mastocytosis and urticaria, but they also play an initiating and enhancing role in the vicious cycles of skin inflammation in atopic dermatitis, allergic contact dermatitis, and psoriasis. Conclusively, MCs represent the “Jekyll and Hyde” of the immune system, being beneficial in host defense while, at the same time, the “bad guy” driving inflammatory disorders. Both sides, however, highlight MCs as a potential target of therapeutic strategies.
